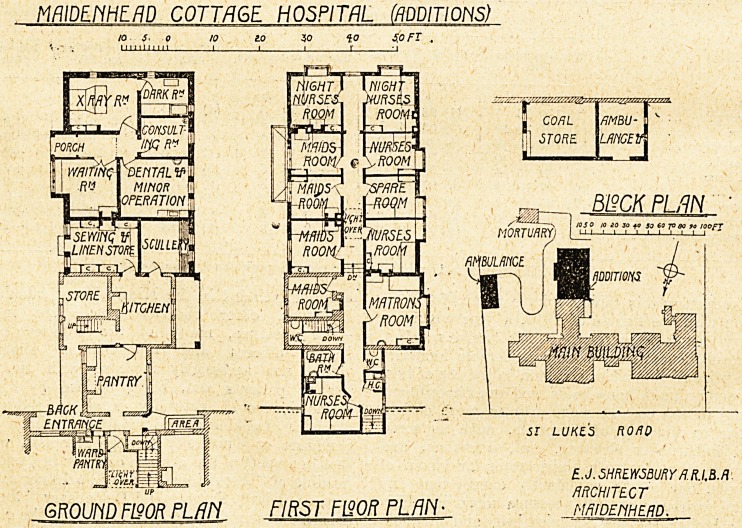# Maidenhead Cottage Hospital

**Published:** 1917-01-13

**Authors:** 


### HOSPITAL ARCHITECTURE AND CONSTRUCTION.

### Maidenhead Cottage Hospital.

This hospital was erected in 1879, and consisted of a symmetrical building in the form of a cross, and having accommodation for eight patients. To realise the extent of the original hospital it is only necessary to blot out the whole of the portion westwards of the letter "N" in "building" together with the part marked "additions." An operation theatre was added in 1889, and the Ada Lewis Wing in 1908.

In the recent alterations the hospital has been extended southwards. The original kitchen is now a pantry, and the former scullery has been converted into the kitchen. Adjoining and communicating with the kitchen is a scullery and a sewing- and linen-store. The former coal-store is now marked "store," and, as there is no other shown, we presume this serves the double purpose of larder and store. The only access except by way of an open verandah to the kitchen and the linen-room is through the pantry, which thus becomes a passage-room, and the only way of getting at the linen-room is through the kitchen. Beyond the linen-room and scullery is a small out-patient department, with separate entrance. This includes a waiting-room, consulting-room, room for minor operations and dental surgery, and an *x*-ray-room with dark-room attached. The upper floor contains the matron's bedroom, six bedrooms for nurses, and four rooms for maids, with a bathroom, w.c., and housemaids' closet. A detached building contains ambulance-house and coal-store. These additions were planned by Mr. E. J. Shrewsbury, A.R.I.B.A., of Maidenhead.

## Figures and Tables

**Figure f1:**